# Effect of Air Injection Depth on Big-bubble Formation in Lamellar Keratoplasty: an Ex Vivo Study

**DOI:** 10.1038/s41598-018-36522-w

**Published:** 2019-03-07

**Authors:** Young-Sik Yoo, Woong-Joo Whang, Min-Ji Kang, Je-Hyung Hwang, Yong-Soo Byun, Geunyoung Yoon, Sungwon Shin, Woonggyu Jung, Sucbei Moon, Choun-Ki Joo

**Affiliations:** 1 Department of Convergence Medical Science, College of Medicine, The Catholic University of Korea, Seoul, South Korea; 2 Department of Ophthalmology and Visual Science, Seoul St. Mary’s Hospital, College of Medicine, The Catholic University of Korea, Seoul, South Korea; 3 Department of Ophthalmology, Sang-gye Paik Hospital, Inje University, Seoul, South Korea; 4 Flaum Eye Institute, The Institute of Optics, Center for Visual Science, University of Rochester, Rochester, New York USA; 5 Department of Biomedical Engineering, Ulsan National Institute of Science and Technology, Ulsan, South Korea; 6 Department of Physics, Kookmin University, Seoul, South Korea

## Abstract

This study evaluated the effect of air injection depth in the big-bubble (BB) technique, which is used for corneal tissue preparation in lamellar keratoplasty. The BB technique was performed on *ex vivo* human corneoscleral buttons using a depth-sensing needle, based on optical coherence tomography (OCT) imaging technology. The needle tip, equipped with a miniaturized OCT depth-sensing probe, was inserted for air injection at a specified depth. Inside the corneal tissue, our needle obtained OCT line profiles, from which residual thickness below the needle tip was measured. Subjects were classified into Groups I, II, III, and IV based on injection depths of 75–80%, 80–85%, 85–90%, and >90% of the full corneal thickness, respectively. Both Type I and II BBs were produced when the mean residual thicknesses of air injection were 109.7 ± 38.0 µm and 52.4 ± 19.2 µm, respectively. Type II BB (4/5) was dominant in group IV. Bubble burst occurred in 1/16 cases of type I BB and 3/16 cases of type II BB, respectively. Injection depth was an important factor in determining the types of BBs produced. Deeper air injection could facilitate formation of Type II BBs, with an increased risk of bubble bursts.

## Introduction

Lamellar keratoplasty (LK) is one of the latest advances in corneal transplantation procedures, which enhances the success rate of keratoplasty while minimizing subsequent complications, compared with conventional penetrating keratoplasty^1–3^. Deep anterior LK (DALK) and endothelial keratoplasty (EK) have become increasingly popular over the past decade, due to advantageous features which penetrating keratoplasty (PKP) cannot provide^4^. However, LK requires additional corneal tissue preparations for separation of corneal layers; these are technically challenging and very important steps for successful LK.

Pneumatic dissection methods provide relatively simple and reliable approaches for separating corneal layers for corneal preparation in DALK and endothelial keratoplasty (EK)^5–7^. In DALK, the corneal stroma, along with the epithelial layer and Bowman’s layer, is separated from Descemet’s membrane (DM). Anwar and Teichmann introduced the big-bubble (BB) technique for this purpose; in this technique, the stroma can be uniformly detached by the air injected by a syringe needle^8^. This technique can be used for pre-Descemet’s endothelial keratoplasty (PDEK), which is a derivative of EK^6–9^. The corneal endothelial layer, including the DM and a portion of the stromal layer, can be manually separated for PDEK.

In cases of EK, the BB technique can be used to make a donor graft by insertion of the needle tip on the endothelial side^8,9^. After placement of a donor corneoscleral button with the endothelial side up, the surgeon holds the corneoscleral button with one hand and performs the BB technique with the other hand, using a 27-gauge or thinner needle. The outcome of this pneumatic dissection depends greatly upon the operating surgeon’s sense, experience, and manual skills. Although Dua *et al*. introduced a specially designed clamp for better handling the donor corneal tissue in PDEK graft preparation^10^, the lack of depth information during the needle operation remains both critical and risky. The situation is worse in DALK cases, where the needle must be inserted into the recipient cornea in an anterior approach, which greatly restricts freedom of operation.

To quantify the injection point, optical coherence tomography (OCT) can be utilized for its depth-resolving imaging capabilities. Pasricha *et al*.^11^ reported the use of a microscope-integrated OCT instrument which could visualize the position of the tip of the injection needle in the corneal tissue; this allowed those investigators to perform depth-controlled injections. They found that deeper corneal air injection led to more successful BB formation in their *ex vivo* study. However, the power of such an OCT instrument is constrained by the optical properties of the injection needle. Because of its opaqueness, the underlying corneal part cannot be imaged with the optical technique. The depth of the needle tip must be found indirectly, in the context of partial images. It is also technically challenging to track the needle motion in the imaging field.

The BB formation is classified into types I and II, depending on where the corneal layer separation is made: either in stroma (type I) or between stroma and DM (type II)^12–15^. The mode of corneal graft preparation for EK can be selected, if it is possible to control the BB type. Typically, a Type II BB involves a large air bubble in its formation^16,17^. It has been reported that Type II BBs are produced at a lower frequency^12,16,18^. By the nature of the BB technique, the BB type is likely to depend on the depth of the air injection point. Here, we hypothesized that quantitative control of the injection point is an important parameter, both for successful BB formation and for achieving the desired type of BB in controlled detachment of corneal layers.

The goal of the present study was to evaluate BB formation by the air injection depth, for the purpose of making corneal grafts for LK. In our research, a needle-type device of real-time depth monitoring was developed to objectively measure the position of the needle tip during insertion, as well as to inject the air for BB formation. By utilizing the smart needle, the effect of the injection depth in the BB technique was quantified using human corneas in an *ex vivo* study.

## Methods

### Subjects

This experimental investigation used human donor corneoscleral buttons obtained from an eyebank (Eversight, Seoul, South Korea). It was conducted with the approval of the Institutional Review Board of Seoul St. Mary’s Hospital (IRB #KC16SISI0795). All methods were performed in accordance with the relevant guidelines and regulations. Corneoscleral buttons were used 1–2 months after being harvested. Their mean thickness was 765.1 ± 51.2 μm. All of the corneoscleral buttons were treated with dextran (DE130, Spectrum Chemical Corp., New Brunswick, NJ, USA) to reduce corneal oedema. After dissolving 10 g of dextran powder in 40 cc of balanced salt solution (BSS Plus^®^, Alcon Laboratories Inc., Fort Worth, TX, USA), the corneoscleral buttons were submerged in dextran solution. All of the corneoscleral buttons were placed with corneal epithelial sides down in the solution and soaked for 30 minutes.

The subjects were divided into four groups, according to the relative depth at which the air was injected into the corneal stromal layer. The depth was measured by the ratio of the depth of the air injection to the full corneal thickness. The depth of the injection point and the corneal thickness were measured by the same OCT system with a needle-type OCT optical probe. The full thickness was obtained by determining the mean thickness of four different points on the mid-peripheral cornea. Groups I, II, III, and IV were defined as the subject groups of 75–80%, 80–85%, 85–90%, and >90% in relative injection depths, respectively. The BB technique was performed using our depth-sensing air injection needle while monitoring the injection depth. The outcome of this procedure was evaluated by the success of the BB formation and the type of BB formed in the subject’s cornea.

### Depth-sensing Needle

**Figure 1:**
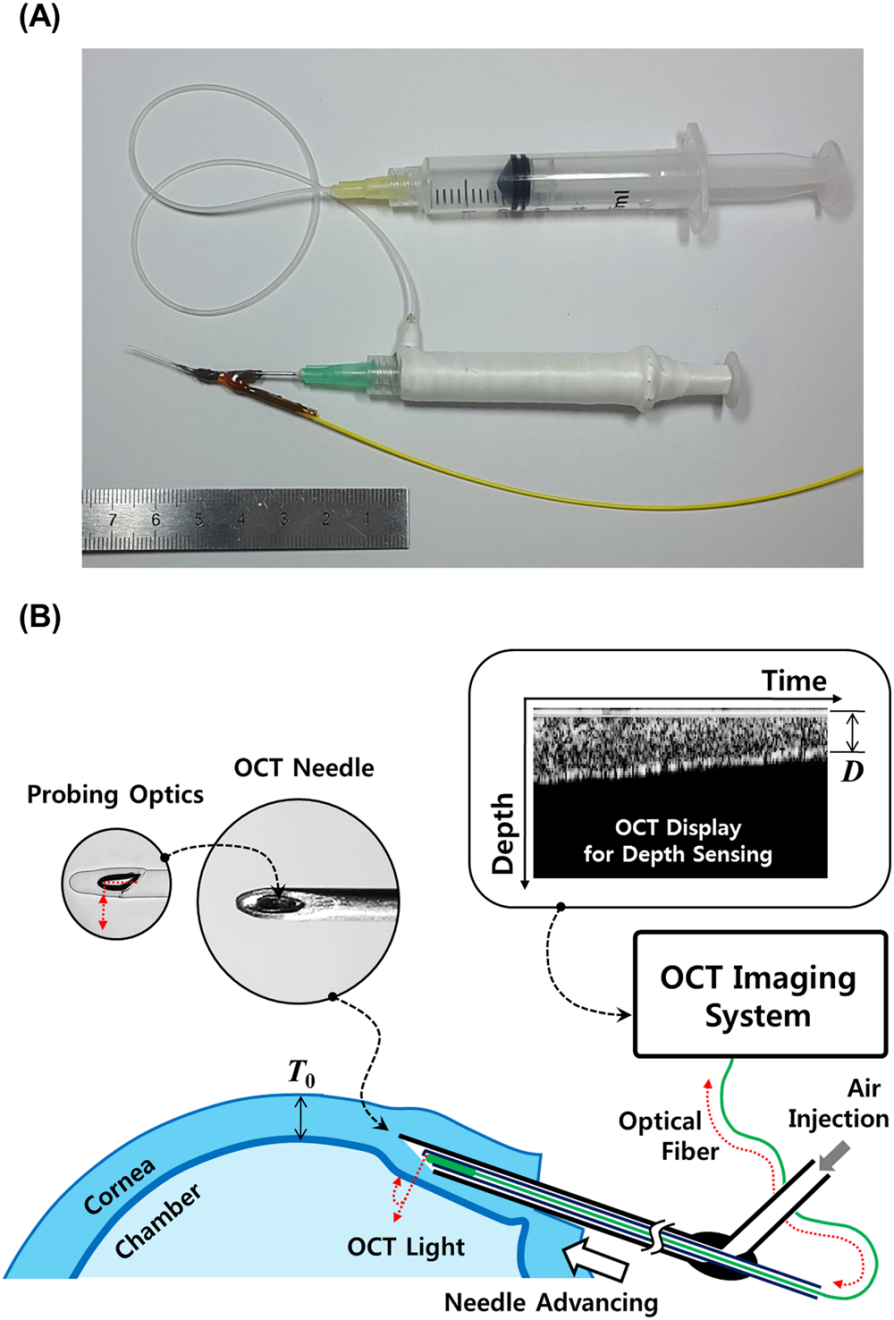
Overall appearance (**A**) and schematic diagram (**B**) of depth-sensing needle is shown. The 26-gauge needle was equipped with an OCT-based depth sensor with an air injection channel.

The depth-sensing needle system was utilized in this study for successful BB formations. Figure 1 shows the schematic diagram of our OCT-based depth-sensing system. Our OCT imaging needle was a 26-gauge injection syringe needle internally equipped with a micro-optical OCT probe^19–21^. Its fully fibre-based design of probing optics was based on stepwise transitional core (STC) fibres in a simple and compact structure^21^. In the form of a thin optical fibre, its outer diameter was only 125 µm, consisting of specialized optical fibres in concatenation and a protective glass cap at the end with a 45°-tilted reflector. The glassy-fibre-optic probe was jacketed for robust operation with steel tubing of 200 µm outer diameter. This reinforced OCT probe was placed and fixed inside the needle’s steel tubing body. OCT light was reflected to the nominally perpendicular direction by the reflector at the distal end. Then, it was illuminated out through the needle’s bevel window. When the needle was inserted into a cornea, the light reflected from the tissue was recollected by the same probing optics, then delivered backward to the main part of the OCT system. Our needle maintained its injection capabilities through the fluidic channel formed between the needle’s tubing body and the outer surface of the steel-jacketed fibre optics. Connected to an injection pump, the airflow channel was split from the optical fibre channel of OCT sensing with no functional interference. The pump was connected to the needle through a flexible silicone tube for the airflow delivery. The pump was operated by the operator’s hand that was opposite to the hand that held the depth-sensing needle. By this two-hand operation scheme, the operator could obtain enhanced precision and maintain increased freedom in the needle work.

### Depth Measurement

By its interferometric operating principle, the OCT imaging system acquired depth-resolved reflection profiles, so called A-lines, from the light recollected by the imaging needle. A swept-source OCT system was used in this study, operating at 1.3 µm for the centre wavelength. The axial resolution was estimated to be 8 μm in corneal tissue (*n* = 1.35). Note that our OCT system differs from ordinary ones in that it equips no beam scanning mechanism to construct image frames. A-lines were continuously acquired in time, which visualized the underlying tissue only on the beam line. Thus, our needle-based system is more appropriately described as a depth-sensing tool than an imaging system. However, an image-like frame could be obtained by stacking the A-lines when a slow advancement of the needle was applied by the operator’s hand. For slow operations, multiple A-lines were averaged to obtain an effective A-line rate (at the display) of 50 lines per second. In the OCT display, each A-line was aligned vertically, and arranged horizontally by the sequence of time. Because the speed of the needle’s motion is not constant, the horizontal axis of the frame must not be interpreted as a spatial dimension. It simply displays changes in A-lines over time. The surgeon could find the thickness of corneal tissue lying right below the needle tip. The residual thickness of the tissue’s inner region, denoted by *D* in Fig. 1, was measured from the current A-line. The depth of the needle tip’s position was obtained from this measurement. For this purpose, the full thickness of the mid-peripheral cornea, denoted by *T*_0_ in Fig. 1, could be measured at four different points, constituting the midpoint between the corneal apex and the limbus, before needle work. Then, the mean value, defined as full corneal thickness (*T*_*m*_), allowed determination of the percentile depth of the needle tip by (1 − *D*/*T*_*m*_) × 100% from the residual thickness of *D*. Here, a slight deviation of the refractive index for each layer was neglected. The air could be injected in a controlled manner based on the depth information obtained from our depth-sensing needle system.

### Surgical Procedures

The corneoscleral button was placed on the pressurized artificial anterior chamber (Moria SA, Antony, France), such that the corneal epithelial layer was “face up.” The artificial anterior chamber was connected to the syringe through an infusion line, and a three-way valve was inserted in the middle of the infusion line. The pressure of the anterior chamber was set between 15 to 20 mmHg, measured with Tono-Pen (Reichert Technologies, Buffalo, NY, USA), and the infusion line was closed by manipulating the three-way valve to maintain the pressure of the anterior chamber. Total corneal thickness was measured at two points (i.e., corneal centre and four mid-peripheral sites (2.0 mm from the centre)) with our customized OCT in the form of a needle. The 300-µm depth of trephination was performed at 8.0 mm diameter using the guarded trephine (Moria SA). Partial corneal incision was made along with the trephination site to easily insert the needle to the corneal stromal layer. Because the needle was marked for orientation on the side opposite to the bevel of the needle, the surgeon typically maintained the needle face down, towards the posterior surface of cornea. When the needle tip arrived at the intended depth, the surgeon opened the infusion line to reduce the pressure in the anterior chamber, and then injected the air to generate a BB. During the entire procedure, the pressure in the artificial anterior chamber was maintained with a constant column height that provided a normal physiologic intraocular pressure for all subjects.

### Statistical Analysis

Statistical analysis was performed using the MS Windows version of SPSS software (version 19.0; SPSS, Inc., Chicago, IL, USA). We performed Wilcoxon rank-sum tests on continuous variables and Fisher’s exact test on categorical variables for univariate analyses. A *P* value of <0.05 was considered statistically significant. All values are presented as mean ± standard deviation or n (%), unless otherwise stated.

## Results

Mean thickness at the mid-peripheral area for 28 corneas in four different groups was 632.9 ± 149.1 µm after treatment with dextran solution. BB was successfully made using a depth-sensing needle in enrolled subjects, except in six cases (Table 1). An incomplete BB was made in two, one, and one cases in groups I, II, and III respectively. Perforation of the corneal endothelial layer occurred in one (1/7, 14.3%) and two (2/7, 28.6%) cases during insertion of the needle tip until it reached the intended depth in groups III and IV, respectively. BB was made in 21 (16 and five of type I and II BB, respectively) cases in all groups. The average residual thickness of air injection was D = 96.0 ± 42.2 µm; it was 109.7 ± 38.0 µm and 52.4 ± 19.2 µm in type I and II BBs, respectively. In four cases of incomplete BB formation, the residual thickness of air injection was D = 144.1 ± 14.0 µm.

**Table 1:** Outcomes of interlayer separation performed by the big-bubble technique, using the depth-sensing needle for different percentile depth ranges.

	Number	Relative Depth of Air Injection^a^ (%)	Interlayer Separation by BB (%)	Incomplete BB Formation^b^ (%)	Perforation Rate by Needle^b^ (%)
Group I	7	77.7 ± 1.3	5 (71.4%)	2 (28.6%)	0 (0%)
Group II	7	81.2 ± 1.4	6 (85.7%)	1 (14.3%)	0 (0%)
Group III	7	87.6 ± 1.2	5 (71.4%)	1 (14.3%)	1 (14.3%)
Group IV	7	91.4 ± 1.1	5 (71.4%)	0 (0%)	2 (28.6%)

^a^Injecting depth was defined as the relative ration for the depth where the air was injected to the total corneal thickness measured before the operation.

^b^Perforation developed in the corneal endothelial layer when the needle tip was inserted into cornea.

BB = big bubble.

Data are presented as number and percentage or mean and standard deviation.

**Figure 2:**
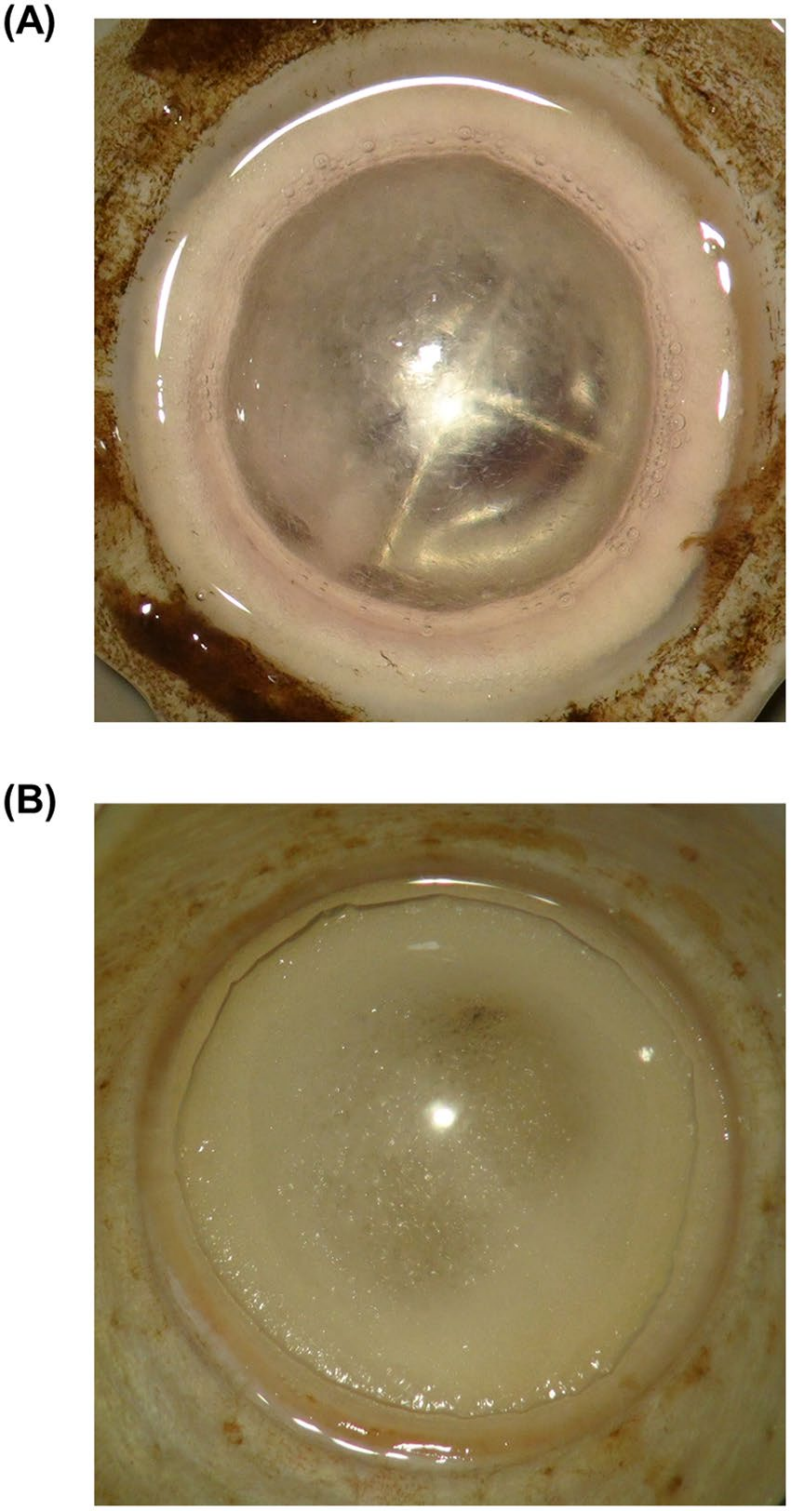
Back surfaces of different types of the big bubbles (BBs) created with the depth-sensing needle. A dome-shaped and relatively small diameter of BB was found in type I BB (**A**), compared with type II BB (**B**).

**Figure 3:**
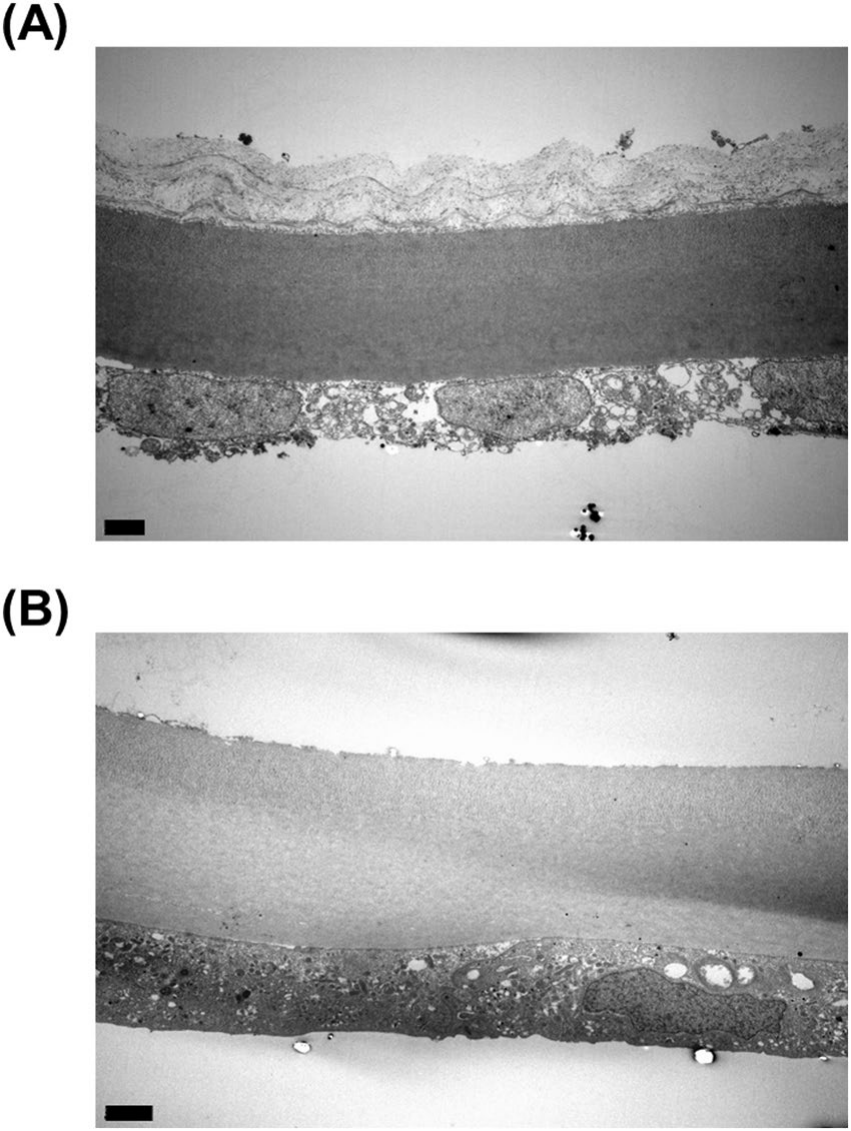
Transmission electron microscopic images for type I and II big bubbles (BB) created by the depth-sensing needle, respectively. Descemet’s membrane and the corneal endothelial layer were found with posterior stromal layer (**A**) or without posterior stromal layer (**B**) in types I and II BBs, respectively. The black solid scale bar represents 2 µm.

For the BBs formed, their shape could be classified into two types, as shown in Fig. 2. Type I BBs (Fig. 2A) had relatively smaller diameters, compared with those of type II BBs (Fig. 2B). Type I (Supplementary Video S1) and II (Supplementary Video S2) BBs were found to be produced by starting from the central and peripheral cornea regions, respectively. Type II BBs comprised large bubbles that spread widely, whereas type I BBs had dome-shaped bubbles. From transmission electron microscopic (TEM) analysis, Type I BBs were found to contain remaining posterior stromal layers (Fig. 3A), while no stromal layers were found in Type II BBs (Fig. 3B).

The overall frequency of BB formation was not strongly affected by the injection depth (Table 2); however, the type of BB exhibited a significant correlation. Type I BBs were obtained commonly in all groups, including Groups I and II; in contrast, Type II BBs were only produced in Groups III and IV, with deeper injection. Type II BBs were obtained in one (1/5, 20.0%) and four cases (4/5, 80.0%) in Groups III and IV, respectively. The diameter of Type II BBs was larger than that of Type I BBs. Type II BBs burst more frequently, as observed in one and two cases in Groups III and IV, respectively.

**Table 2:** Big-bubble formation by producing big bubbles in human cornea, according to the different depths for injection of air with the depth-sensing needle.

	Depth of Air Injection^a^ (µm)	BB Type		BB Diameter (mm)		Bursting BB^b^	
		Type I	Type II	Type I	Type II	Type I	Type II
Group I	45.9 ± 8.8	5	0	7.65	N/A	0	N/A
Group II	89.6 ± 27.6	6	0	8.30	N/A	0	N/A
Group III	110.6 ± 23.3	4	1	8.61	10.21	1	1
Group IV	150.0 ± 17.3	1	4	8.71	10.15	0	2

^a^The corneal thickness presented in the optical coherence tomography image cannot provide an absolute value for the actual corneal thickness, due to the deviation of the refractive index for each corneal layer and each person.

^b^Perforation developed when a BB was made by inserting air into corneal stroma.

BB = big bubble.

Data are presented as number and percentage or median and range.

## Discussion

The present study demonstrated the effect of air injection depth on BB formation. We observed that the type of BB was affected by the injection depth during its formation. Type II BBs could be obtained by locating the injection point closer to DM. However, the incidences of perforations in corneal endothelial layers and of formed bubbles bursting during the air injection were also increased. In most previous studies conducted by other groups, the injection point was identified as an important factor, which must be close to DM for successful BB formation^22–24^. However, information regarding exact distance was not provided in most of these reports. Pasricha *et al*.^11^ reported that the average relative depth was 79.9 ± 3.0% for successful BB formation, regardless of the type of BB, in their *ex vivo* experiment with a microscope-integrated OCT system. In our study, the results showed that very deep injection was not necessary for BB formation, as long as the depth exceeded 75%. However, formation of a specific BB type could be controlled by the injection depth. This property will be very useful for surgeons who expect a certain type of BB for their procedures.

Other factors may affect the type of BB used in the BB technique. In the clinical application of DALK, Goweida reported that the frequency of making Type I BB (56/72, 77.8%) was higher than that of Type II BB (14/72, 19.4%), regardless of the surgeon’s intention^18^. Age of the patient, presence of deep corneal scars, and corneal thickness were identified as considerable preoperative factors associated with Type II BB formation. Although they did not clearly specify the types of BBs, Feiz *et al*.^25^, reported that male patients and large trephination sizes increased the rate at which detached bare DM was achieved in DALK. Furthermore, in prior reports of *ex vivo* studies of the BB technique, the rate of Type II BB formation was relatively low, ranging from 6.3% to 23.8%^12,16,26^. The results of the experimental study by Dua *et al*. are comparable to those of our present study^16,26^. In their study, a needle was inserted radially, from the scleral rim to mid-peripheral cornea at mid-stromal depth, while a corneoscleral button was placed with the endothelial side up. After carefully observing movement of the injected air inside the corneal stroma, those investigators concluded that clusters of fenestrations present in the periphery of the pre-Descemet’s layer might play an important role in producing Type II BBs. These clusters of fenestrations were analysed to determine the path of the injected air to a plane between the pre-Descemet’s layer and DM. In the report by AlTaan *et al*.^27^, the posterior walls of Type I BBs were reported as thicker than those of Type II BBs, on the basis of OCT images. The previous studies suggest that the actual cleavage plane of detachment made by the BB technique could be located at deeper corneal stroma than that at which the injection point of the needle tip is located. This explains one case of Type II BB in Group III, observed in our experiment. However, the overall chances of forming Type II BBs must have increased by reducing the distance of the injection point to DM. Although the mechanism of Type II BB formation is not clearly explained by the present study, or in conjunction with the previous findings, it seems intuitively reasonable to assume that deeper injection is beneficial for the generation of thin-wall Type II BBs.

A relatively high incidence of perforation was observed in Group IV, with deeper injection. This is partly explained by the dominance of Type II BBs in Group IV; these are naturally more vulnerable, due to their thin walls. Other factors might contribute including the structure of our probe-equipped needle. The optical probing point in our needle was not the very end of the needle tip; rather, it was located 0.7 mm axially from the tip. When the tip rapidly penetrated the residual corneal layers, the information obtained from the OCT optics might not have reflected the current status. Such sudden failure was produced especially in associated with corneal wrinkling induced by advancement of the needle in the stroma. For this reason, all perforations in our experiment occurred when handling the needle. Improvement of needle size, structure, and material composition might reduce this complication in the future.

Our depth-sensing needle was based on the optical imaging technology of OCT. The depth information was obtained from the optical reflectance profile of the laser beam, which was fired out from the miniaturized optical probe equipped inside the needle body. Compared with the microscope-integrated OCT system that others have utilized for an advanced BB technique^11,28–30^, our needle tool is advantageous with respect to its operational and instrumentational simplicity. Depth measurement can be performed on targeted tissue with the injection needle, *in situ* and in real time, without interpretational delays. However, our methods have some limitations and error sources. First, our optical sensor measures thickness, rather than depth. In our study, the relative depth was obtained by normalizing the acquired residual thickness by the full thickness of cornea, which was separately measured. Thus, non-uniformity of corneal thickness might produce errors in the measured relative depth. We consider this to be a critical issue in practice. Note that it is not yet possible to determine whether relative depth is more significant than residual thickness. Inconsistency between those two different measures must be sufficiently small for typical cases where corneal thickness generally does not vary. Second, geometric measurement errors could be produced when the fired laser beam was not oriented in a direction normal to the corneal plane. The beam’s angle to the normal direction might make our OCT system overestimate residual thickness. As the measurement is governed by a cosine function of the angle, the expected measurement errors rarely exceed 6% for the maximum angular deviation of the needle below 20 degrees. We minimized this effect by ensuring careful handling by the surgeon during the procedure, using an orientation mark on the needle. Note that ordinary OCT imaging techniques may also exhibit such geometric inaccuracies caused by beam refraction on the curved corneal surface.

It is worth noting that the surgeon utilized microscopic views, as well as the information provided by our needle-based OCT system, for depth monitoring in our experiment. We observed a crescent-shaped thin dark line (as marked with yellow arrows in Supplementary Fig. S3) that appeared in front of the needle tip when it reached a depth near that of DM. This observation was consistent with that of Scorcia *et al*.^31^. We suspect that the pattern was produced by the thin tissue layers minutely wrinkled or stressed by the advancement of the needle. Along with the OCT information, the crescent-shaped pattern was a useful indicator that deep positions had been reached. Although these types of features can be useful in the BB technique, they generally do not provide quantitative information and may depend on a variety of factors to maintain reliability.

Our study demonstrated that the depth information obtained in the BB technique could provide multiple benefits in corneal preparations for LK. The depth-controlled injection would make the BB technique more manageable and reproducible with respect to the resulting BB type; it would also minimize undesirable complications. For clinical applications, there remain some challenges to resolve, including an appropriate method to excise the endothelial graft with a circular shape after BB formation, while causing minimal endothelial cell damage. We believe that image-guided injection, such as our method, can advance corneal preparation procedures through further experimental and clinical studies.

## Electronic supplementary material

Supplement Video and Figures

Supplementary Video S1

Supplementary Video S2
