# Supplementary material for: Effect of Air Injection Depth on Big-bubble Formation in Lamellar Keratoplasty: an Ex Vivo Study
Source: Sci Rep. 2019 Mar 7;9:3785. doi: 10.1038/s41598-018-36522-w (PMC6405887; doi:10.1038/s41598-018-36522-w)
Supplement: Supplementary file 1 — Supplement Video and Figures [file 41598_2018_36522_MOESM1_ESM.pdf]

Effect of Air Injection Depth on Big-bubble Formation in Lamellar Keratoplasty: an Ex Vivo Study

Young-Sik Yoo, MD[1], Woong-Joo Whang[2], Min-Ji Kang[2], Je-Hyung Hwang[3], Yong-Soo Byun[2], Geunyoung Yoon[4], Sung-Won Shin[5], Woong-Gyu Jung[5], Sucbei Moon[6*] and Choun-Ki Joo, MD, PhD[2*]

Supplementary Video S1. The video shows a type 1 big bubble (BB) made using a depth-sensing needle in a human corneoscleral button placed on a pressurized artificial anterior chamber. When the BB was made, it was produced in a central to peripheral manner.

Supplementary Video S2. The video shows a type 2 big bubble (BB) made using depth-sensing needle in a human corneoscleral button placed on a pressurized artificial anterior chamber. When the BB was made, it was produced in a peripheral to central manner.

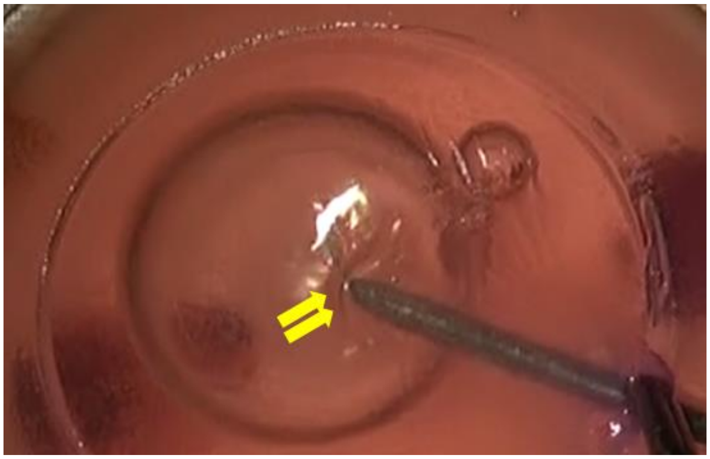

Supplement Figure S3. Crescent-shaped dark line (marked with yellow arrow) found on the surgical microscope view during needle insertion into the cornea. This signature may help the surgeon recognize a large depth of the needle tip's position, serving as a secondary method of depth sensing.
